# Methyl 4-{[6-(4-bromo­phen­yl)-3-oxo-2,3,4,5-tetra­hydro­pyridazin-4-yl]methyl}benzoate

**DOI:** 10.1107/S160053681101467X

**Published:** 2011-04-29

**Authors:** Adailton J. Bortoluzzi, Luciana B. P. Souza, Antônio C. Joussef, Emerson Meyer

**Affiliations:** a Departamento de Química, UFSC, 88040-900 Florianópolis, SC, Brazil; b Departamento de Química, UEM, 87020-900 Maringá, PR, Brazil

## Abstract

The structure of the title compound, C_19_H_17_BrN_2_O_3_, consists of two cyclic groups, *viz.* 4-(meth­oxy­carbon­yl)phenyl and 6-(4-bromo­phen­yl)-3-oxo-2,3,4,5-dihydro­pyridazin-4-yl, which are linked by a methyl­ene spacer. The pyridazine ring is twisted and the dihedral angle between its mean plane and that of the bromo­phenyl mean plane is 17.2 (2)°. The 4-(meth­oxy­carbon­yl)phenyl group shows a quasi-planar conformation, where the dihedral angle between the mean planes of the phenyl ring and carboxyl­ate ester group is 7.9 (4)°. Centrosymmetric inter­molecular N—H⋯O hydrogen bonds form dimers. These are linked by C—Br⋯O=C inter­actions [Br⋯O  = 3.10 (1) Å] to form a one-dimensional polymeric structure running along the [1
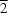
0] direction.

## Related literature

For specific details concerning organic reactions and synthetic procedures for 4,5-diihydro-3(2*H*)-pyridazinone derivatives, see: Meyer *et al.* (2004 ▶). For the biological activity of heterocyclic compounds containing the 3(2*H*)-pyridazinone group, see: Sayed *et al.* (2002 ▶); Katrusiak & Baloniak (1994 ▶); Dogruer *et al.* (2003 ▶); Pieretti *et al.* (2006 ▶); Cao *et al.* (2003 ▶); Piaz *et al.* (1994 ▶); Xu *et al.* (2008 ▶); Giovannoni *et al.* (2007 ▶); Coelho *et al.* (2007 ▶); Malinka *et al.* (2003 ▶); Wexler *et al.* (1996 ▶); Barbaro *et al.*, (2001 ▶); Vergelli *et al.* (2007 ▶); Abudshait (2007 ▶). For related structures, see: Zhang *et al.* (2006 ▶); Zhou & Zhou (2007 ▶). For C—Br⋯O inter­actions, see: Voronina *et al.* (2009 ▶)
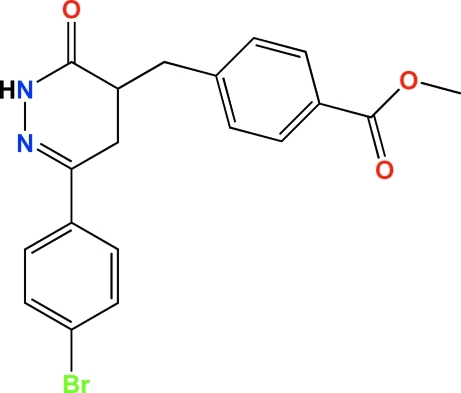


## Experimental

### 

#### Crystal data

C_19_H_17_BrN_2_O_3_
*M* *_r_* = 401.26
Triclinic, 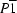

*a* = 5.991 (1) Å
*b* = 8.958 (1) Å
*c* = 17.531 (2) Å
α = 99.502 (11)°
β = 95.241 (12)°
γ = 105.499 (10)°
*V* = 885.1 (2) Å^3^
*Z* = 2
Mo *K*α radiation
μ = 2.34 mm^−1^
*T* = 293 K
0.50 × 0.33 × 0.13 mm

#### Data collection

Enraf–Nonius CAD-4 diffractometer
Absorption correction: ψ scan [North *et al.* (1968 ▶) and *PLATON* (Spek, 2009 ▶)] *T*_min_ = 0.567, *T*_max_ = 0.978
3368 measured reflections
3151 independent reflections
2033 reflections with *I* > 2σ(*I*)
*R*_int_ = 0.024
3 standard reflections every 200 reflections  intensity decay: 1%

#### Refinement

*R*[*F* ^2^ > 2σ(*F* ^2^)] = 0.038
*wR*(*F* ^2^) = 0.098
*S* = 1.03
3151 reflections
237 parameters
4 restraints
H-atom parameters constrained
Δρ_max_ = 0.28 e Å^−3^
Δρ_min_ = −0.36 e Å^−3^

### 

Data collection: *CAD-4 Software* (Enraf–Nonius, 1989 ▶); cell refinement: *SET4* in *CAD-4 Software*; data reduction: *HELENA* (Spek, 1996 ▶); program(s) used to solve structure: *SIR97* (Altomare *et al.*, 1999 ▶); program(s) used to refine structure: *SHELXL97* (Sheldrick, 2008 ▶); molecular graphics: *PLATON* (Spek, 2009 ▶) and *Mercury* (Macrae *et al.*, 2008 ▶); software used to prepare material for publication: *SHELXL97*.

## Supplementary Material

Crystal structure: contains datablocks global, I. DOI: 10.1107/S160053681101467X/lw2061sup1.cif

Structure factors: contains datablocks I. DOI: 10.1107/S160053681101467X/lw2061Isup2.hkl

Supplementary material file. DOI: 10.1107/S160053681101467X/lw2061Isup3.mol

Additional supplementary materials:  crystallographic information; 3D view; checkCIF report

## Figures and Tables

**Table 1:** Hydrogen-bond geometry (Å, °)

*D*—H⋯*A*	*D*—H	H⋯*A*	*D*⋯*A*	*D*—H⋯*A*
N3—H3⋯O1^i^	0.86	2.08	2.910 (4)	162

Symmetry codes: (i) 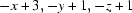; (ii) 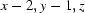.

**Fig. 1.**
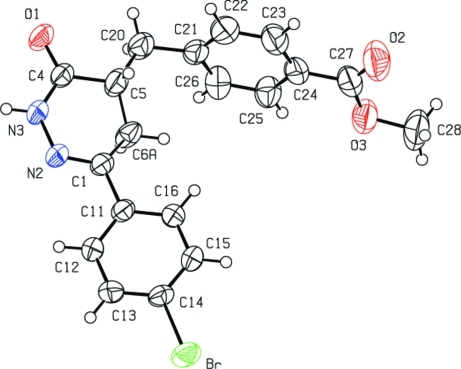
The molecular structure of title compound showing the atom-labelling scheme. Ellipsoids are drawn at the 50% probability level.

**Fig. 2.**
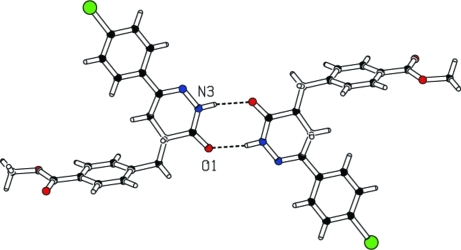
Dimeric structure formed by hydrogen bonds. Symmetry code: -x + 3, -y + 1, -z + 1

**Fig. 3.**
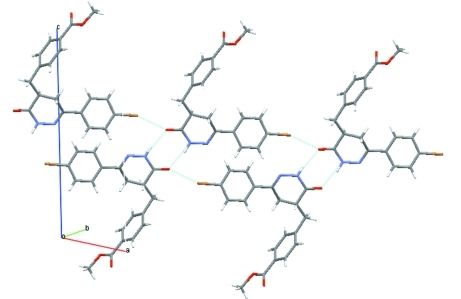
One-dimensional polymeric structure formed by C—Br···O=C interactions. Symmetry code: -2 + x, -1 + y, z

**Fig. 4.**
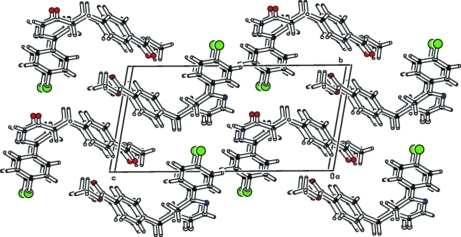
Packing showing the molecules staked along [100] direction.

## supplementary crystallographic information

### Comment

Heterocyclic compounds containing 3-(2*H*)-pyridazinone moiety in their structures have attracted a great deal of attention due to their wide spectrum of biological activity such as antimicrobial (Sayed *et al.*, 2002; Katrusiak & Baloniak, 1994), anti-inflammatory (Dogruer *et al.*, 2003; Pieretti *et al.*, 2006), antifeedant (Cao *et al.*, 2003), herbicidal (Piaz *et al.*, 1994; Xu *et al.*, 2008), antiplatelet (Giovannoni *et al.*, 2007; Coelho *et al.*, 2007), anticancer (Malinka *et al.*, 2003), antihypertensive (Wexler *et al.*, 1996; Barbaro *et al.*, 2001), antinociceptive agent (Giovannoni *et al.*, 2007; Vergelli *et al.*, 2007) and other biological and pharmacological properties (Abudshait, 2007). In our study toward the synthesis of dihydropyridazinones as potential candidates for antihypertensive activity the structure of methyl 4-[6-(4-bromophenyl)-3-oxo-2,3,4,5-dihydropyridazin-4-ylmethyl]benzoate has been determined.

The structure of the title compound consists of two cyclic moieties, 4-(methoxycarbonyl)phenyl and 6-(4-bromophenyl)-3-oxo-2,3,4,5-dihydropyridazin-4-yl, which are linked by methylene spacer (Fig. 1). The pyridazinyl ring is twisted, the greatest deviation is observed for carbon atoms C5 and the disordered C6A and C6B atoms, which are -0.0674 (8), 0.479 (5) and -0.415 (12) Å, respectively, out of the mean plane of all atoms in the ring. The dihedral angle between the mean plane of this ring and that of the bromophenyl mean plane is 17.2 (2)°. The 4-(Methoxycarbonyl)phenyl moiety shows quasi-planar conformation, where the dihedral angle between the mean planes of the phenyl ring and carboxylate ester group is 7.9 (4)°. Intermolecular N3—H3N···O1 hydrogen bonds form centrosymmetric dimers (Fig. 2). Each dimer is linked to two neighboring dimers through C4═O1···Br—C14 interactions (Voronina *et al.*, 2009) forming an one-dimensional polymeric structure along [120] direction (Fig. 3). In addition, packing analysis shows that the molecules are perfectly stacked along [100] direction (Fig. 4).

### Experimental

The title compound was synthesized according to a previously described method (Meyer *et al.*, 2004). A careful crystallization from methanol/water (1:1 v/v) provided colorless crystals suitable for X-ray analysis.

### Refinement

H atoms were placed at their idealized positions with distances of 0.93, 0.98, 0.97 and 0.96 Å and *U*_iso_ fixed at 1.2 and 1.5 times *U*_eq_ of the preceding atom for C—H_Ar_, CH, CH_2_ and CH_3_, respectively. H atom bonded to N atom at the pyridazinyl ring was found from Fourier difference map and treated with riding model and its *U*_iso_ fixed at 1.2 times *U*_eq_ of the parent atom. One C atom (C6) of the pyridazinyl ring is disordered over two alternative positions. The position of the disordered atoms were restrained and the occupancies were refined giving 0.696 (16) and 0.304 (16) for C6A and C6B, respectively.

### Crystal data

**Table:**

C_19_H_17_BrN_2_O_3_	*Z* = 2
*M**_r_* = 401.26	*F*(000) = 408
Triclinic, *P*1	*D*_x_ = 1.506 Mg m^−^^3^
Hall symbol: -P 1	Mo *K*α radiation, λ = 0.71069 Å
*a* = 5.991 (1) Å	Cell parameters from 25 reflections
*b* = 8.958 (1) Å	θ = 8.2–13.4°
*c* = 17.531 (2) Å	µ = 2.34 mm^−^^1^
α = 99.502 (11)°	*T* = 293 K
β = 95.241 (12)°	Block, colourless
γ = 105.499 (10)°	0.50 × 0.33 × 0.13 mm
*V* = 885.1 (2) Å^3^	

### Data collection

**Table:**

Enraf–Nonius CAD-4 diffractometer	2033 reflections with *I* > 2σ(*I*)
Radiation source: fine-focus sealed tube	*R*_int_ = 0.024
graphite	θ_max_ = 25.1°, θ_min_ = 2.4°
ω/2θ scans	*h* = −7→6
Absorption correction: ψ scan [*PLATON* (Spek, 2009) and North *et al.* (1968)]	*k* = 0→10
*T*_min_ = 0.567, *T*_max_ = 0.978	*l* = −20→20
3368 measured reflections	3 standard reflections every 200 reflections
3151 independent reflections	intensity decay: 1%

### Refinement

**Table:**

Refinement on *F*^2^	Primary atom site location: structure-invariant direct methods
Least-squares matrix: full	Secondary atom site location: difference Fourier map
*R*[*F*^2^ > 2σ(*F*^2^)] = 0.038	Hydrogen site location: inferred from neighbouring sites
*wR*(*F*^2^) = 0.098	H-atom parameters constrained
*S* = 1.03	*w* = 1/[σ^2^(*F*_o_^2^) + (0.047*P*)^2^ + 0.1645*P*] where *P* = (*F*_o_^2^ + 2*F*_c_^2^)/3
3151 reflections	(Δ/σ)_max_ < 0.001
237 parameters	Δρ_max_ = 0.28 e Å^−^^3^
4 restraints	Δρ_min_ = −0.36 e Å^−^^3^

### Fractional atomic coordinates and isotropic or equivalent isotropic displacement parameters (Å^2^)

**Table:**

	*x*	*y*	*z*	*U*_iso_*/*U*_eq_	Occ. (<1)
Br	−0.15083 (6)	−0.19522 (5)	0.35018 (3)	0.06171 (18)	
O1	1.4961 (4)	0.5375 (3)	0.40556 (15)	0.0636 (7)	
O2	0.7322 (7)	0.1015 (5)	−0.0912 (2)	0.1113 (14)	
O3	0.5072 (5)	0.2549 (4)	−0.05798 (17)	0.0862 (10)	
N2	0.9579 (5)	0.2989 (3)	0.44336 (17)	0.0477 (7)	
N3	1.1821 (5)	0.4010 (3)	0.45053 (17)	0.0495 (7)	
H3	1.2594	0.4324	0.4969	0.059*	
C1	0.8308 (5)	0.2698 (4)	0.37748 (19)	0.0441 (9)	
C4	1.2910 (6)	0.4559 (4)	0.3928 (2)	0.0478 (9)	
C5	1.1519 (6)	0.4051 (6)	0.3128 (2)	0.0748 (13)	
H5A	1.1718	0.3001	0.2959	0.090*	0.696 (16)
H5B	1.0661	0.4828	0.3257	0.090*	0.304 (16)
C6A	0.9009 (7)	0.3670 (12)	0.3173 (5)	0.053 (2)	0.696 (16)
H61A	0.8150	0.3097	0.2667	0.063*	0.696 (16)
H62A	0.8582	0.4644	0.3297	0.063*	0.696 (16)
C6B	0.936 (2)	0.2760 (19)	0.3030 (5)	0.049 (5)	0.304 (16)
H61B	0.9684	0.1765	0.2852	0.059*	0.304 (16)
H62B	0.8234	0.2891	0.2630	0.059*	0.304 (16)
C11	0.5908 (5)	0.1601 (4)	0.3696 (2)	0.0430 (8)	
C12	0.4841 (6)	0.1357 (4)	0.4353 (2)	0.0485 (9)	
H12	0.5620	0.1913	0.4843	0.058*	
C13	0.2661 (6)	0.0313 (4)	0.4295 (2)	0.0505 (9)	
H13	0.1970	0.0163	0.4741	0.061*	
C14	0.1509 (5)	−0.0509 (4)	0.3570 (2)	0.0459 (9)	
C15	0.2488 (6)	−0.0279 (4)	0.2906 (2)	0.0505 (9)	
H15	0.1680	−0.0826	0.2418	0.061*	
C16	0.4696 (6)	0.0776 (4)	0.2968 (2)	0.0489 (9)	
H16	0.5370	0.0932	0.2520	0.059*	
C20	1.2585 (6)	0.4905 (5)	0.2539 (2)	0.0636 (11)	
H20A	1.4172	0.4836	0.2532	0.076*	
H20B	1.2664	0.6013	0.2686	0.076*	
C21	1.1228 (6)	0.4253 (5)	0.1732 (2)	0.0578 (10)	
C22	1.1646 (7)	0.3022 (6)	0.1252 (3)	0.0761 (13)	
H22	1.2879	0.2642	0.1408	0.091*	
C23	1.0265 (8)	0.2329 (6)	0.0538 (3)	0.0768 (13)	
H23	1.0584	0.1494	0.0220	0.092*	
C24	0.8429 (7)	0.2863 (5)	0.0295 (2)	0.0592 (10)	
C25	0.8033 (7)	0.4128 (5)	0.0761 (2)	0.0659 (11)	
H25	0.6828	0.4528	0.0598	0.079*	
C26	0.9426 (8)	0.4809 (5)	0.1475 (2)	0.0679 (12)	
H26	0.9136	0.5663	0.1787	0.082*	
C27	0.6928 (8)	0.2035 (6)	−0.0460 (3)	0.0723 (12)	
C28	0.3469 (9)	0.1770 (7)	−0.1296 (3)	0.116 (2)	
H28A	0.2053	0.2075	−0.1280	0.173*	
H28B	0.3113	0.0645	−0.1344	0.173*	
H28C	0.4184	0.2075	−0.1736	0.173*	

### Atomic displacement parameters (Å^2^)

**Table:**

	*U*^11^	*U*^22^	*U*^33^	*U*^12^	*U*^13^	*U*^23^
Br	0.0414 (2)	0.0561 (3)	0.0743 (3)	−0.00418 (16)	0.00693 (18)	0.00672 (19)
O1	0.0418 (14)	0.0744 (18)	0.0551 (17)	−0.0138 (13)	−0.0059 (12)	0.0156 (14)
O2	0.117 (3)	0.150 (3)	0.065 (2)	0.070 (3)	−0.010 (2)	−0.025 (2)
O3	0.080 (2)	0.117 (3)	0.058 (2)	0.046 (2)	−0.0112 (16)	−0.0057 (18)
N2	0.0352 (15)	0.0527 (18)	0.0452 (19)	0.0005 (13)	−0.0002 (14)	0.0052 (14)
N3	0.0373 (15)	0.0555 (18)	0.0426 (18)	−0.0010 (13)	−0.0034 (13)	0.0021 (15)
C1	0.0352 (18)	0.049 (2)	0.046 (2)	0.0084 (16)	0.0029 (17)	0.0105 (17)
C4	0.0378 (19)	0.052 (2)	0.046 (2)	0.0031 (16)	−0.0008 (17)	0.0083 (18)
C5	0.049 (2)	0.103 (3)	0.049 (3)	−0.016 (2)	−0.0062 (19)	0.021 (2)
C6A	0.041 (3)	0.051 (5)	0.060 (4)	−0.004 (3)	−0.004 (3)	0.027 (4)
C6B	0.051 (8)	0.041 (9)	0.045 (8)	−0.001 (7)	−0.003 (6)	0.010 (7)
C11	0.0337 (17)	0.047 (2)	0.046 (2)	0.0069 (15)	0.0014 (16)	0.0103 (17)
C12	0.0414 (19)	0.055 (2)	0.040 (2)	0.0050 (17)	0.0025 (16)	0.0011 (17)
C13	0.0406 (19)	0.055 (2)	0.051 (2)	0.0053 (17)	0.0113 (17)	0.0082 (19)
C14	0.0328 (17)	0.045 (2)	0.055 (2)	0.0027 (15)	0.0058 (16)	0.0088 (18)
C15	0.0422 (19)	0.054 (2)	0.044 (2)	0.0036 (17)	−0.0019 (17)	−0.0005 (18)
C16	0.0420 (19)	0.061 (2)	0.040 (2)	0.0066 (17)	0.0063 (16)	0.0113 (18)
C20	0.049 (2)	0.073 (3)	0.057 (3)	−0.005 (2)	0.0006 (19)	0.020 (2)
C21	0.045 (2)	0.071 (3)	0.050 (3)	−0.0007 (19)	0.0047 (19)	0.021 (2)
C22	0.059 (3)	0.111 (4)	0.064 (3)	0.035 (3)	0.006 (2)	0.017 (3)
C23	0.078 (3)	0.102 (4)	0.054 (3)	0.040 (3)	0.009 (2)	0.003 (3)
C24	0.060 (2)	0.078 (3)	0.042 (2)	0.022 (2)	0.0082 (19)	0.016 (2)
C25	0.070 (3)	0.071 (3)	0.056 (3)	0.024 (2)	−0.001 (2)	0.013 (2)
C26	0.082 (3)	0.062 (3)	0.053 (3)	0.016 (2)	−0.001 (2)	0.008 (2)
C27	0.079 (3)	0.095 (4)	0.044 (3)	0.031 (3)	0.009 (2)	0.009 (3)
C28	0.102 (4)	0.165 (6)	0.068 (4)	0.056 (4)	−0.028 (3)	−0.018 (4)

### Geometric parameters (Å, °)

**Table:**

Br—C14	1.901 (3)	C12—C13	1.372 (5)
Br—O1^i^	3.096 (2)	C12—H12	0.9300
O1—C4	1.229 (4)	C13—C14	1.376 (5)
O2—C27	1.196 (5)	C13—H13	0.9300
O3—C27	1.325 (5)	C14—C15	1.372 (5)
O3—C28	1.456 (5)	C15—C16	1.388 (4)
N2—C1	1.271 (4)	C15—H15	0.9300
N2—N3	1.389 (4)	C16—H16	0.9300
N3—C4	1.343 (4)	C20—C21	1.505 (5)
N3—H3	0.8600	C20—H20A	0.9700
C1—C11	1.488 (4)	C20—H20B	0.9700
C1—C6A	1.495 (4)	C21—C22	1.366 (6)
C1—C6B	1.504 (5)	C21—C26	1.375 (6)
C4—C5	1.498 (5)	C22—C23	1.385 (6)
C5—C6B	1.462 (5)	C22—H22	0.9300
C5—C6A	1.463 (4)	C23—C24	1.373 (5)
C5—C20	1.477 (5)	C23—H23	0.9300
C5—H5A	0.9800	C24—C25	1.371 (6)
C5—H5B	0.9800	C24—C27	1.487 (6)
C6A—H5B	1.2052	C25—C26	1.386 (6)
C6A—H61A	0.9700	C25—H25	0.9300
C6A—H62A	0.9700	C26—H26	0.9300
C6B—H61B	0.9700	C28—H28A	0.9600
C6B—H62B	0.9700	C28—H28B	0.9600
C11—C12	1.388 (5)	C28—H28C	0.9600
C11—C16	1.391 (5)		
			
C14—Br—O1^i^	152.32 (12)	C12—C13—C14	119.2 (3)
C27—O3—C28	116.1 (4)	C12—C13—H13	120.4
C1—N2—N3	116.8 (3)	C14—C13—H13	120.4
C4—N3—N2	127.0 (3)	C15—C14—C13	121.0 (3)
C4—N3—H3	116.5	C15—C14—Br	120.3 (3)
N2—N3—H3	116.5	C13—C14—Br	118.7 (3)
N2—C1—C11	116.9 (3)	C14—C15—C16	119.4 (3)
N2—C1—C6A	120.7 (4)	C14—C15—H15	120.3
C11—C1—C6A	121.2 (3)	C16—C15—H15	120.3
N2—C1—C6B	121.5 (7)	C15—C16—C11	120.5 (3)
C11—C1—C6B	115.6 (4)	C15—C16—H16	119.7
O1—C4—N3	121.0 (3)	C11—C16—H16	119.7
O1—C4—C5	122.8 (3)	C5—C20—C21	112.3 (3)
N3—C4—C5	116.1 (3)	C5—C20—H20A	109.1
C6B—C5—C20	129.0 (5)	C21—C20—H20A	109.1
C6A—C5—C20	122.3 (4)	C5—C20—H20B	109.1
C6B—C5—C4	116.4 (6)	C21—C20—H20B	109.1
C6A—C5—C4	110.8 (4)	H20A—C20—H20B	107.9
C20—C5—C4	114.6 (3)	C22—C21—C26	117.9 (4)
C6A—C5—H5A	101.8	C22—C21—C20	121.1 (4)
C20—C5—H5A	101.8	C26—C21—C20	120.9 (4)
C4—C5—H5A	101.8	C21—C22—C23	121.2 (4)
C6B—C5—H5B	90.6	C21—C22—H22	119.4
C20—C5—H5B	90.6	C23—C22—H22	119.4
C4—C5—H5B	90.6	C24—C23—C22	120.6 (4)
H5A—C5—H5B	156.6	C24—C23—H23	119.7
C5—C6A—C1	112.7 (4)	C22—C23—H23	119.7
C1—C6A—H5B	126.3	C25—C24—C23	118.8 (4)
C5—C6A—H61A	109.1	C25—C24—C27	122.6 (4)
C1—C6A—H61A	109.1	C23—C24—C27	118.7 (4)
H5B—C6A—H61A	123.3	C24—C25—C26	120.0 (4)
C5—C6A—H62A	109.1	C24—C25—H25	120.0
C1—C6A—H62A	109.1	C26—C25—H25	120.0
H61A—C6A—H62A	107.8	C21—C26—C25	121.5 (4)
C5—C6B—C1	112.2 (5)	C21—C26—H26	119.2
C5—C6B—H61B	109.2	C25—C26—H26	119.2
C1—C6B—H61B	109.2	O2—C27—O3	123.1 (4)
C5—C6B—H62B	109.2	O2—C27—C24	124.5 (4)
C1—C6B—H62B	109.2	O3—C27—C24	112.4 (4)
H61B—C6B—H62B	107.9	O3—C28—H28A	109.5
C12—C11—C16	118.3 (3)	O3—C28—H28B	109.5
C12—C11—C1	120.5 (3)	H28A—C28—H28B	109.5
C16—C11—C1	121.2 (3)	O3—C28—H28C	109.5
C13—C12—C11	121.4 (3)	H28A—C28—H28C	109.5
C13—C12—H12	119.3	H28B—C28—H28C	109.5
C11—C12—H12	119.3		
			
C1—N2—N3—C4	−9.7 (5)	C12—C13—C14—Br	179.9 (3)
N3—N2—C1—C11	179.8 (3)	O1^i^—Br—C14—C15	137.0 (3)
N3—N2—C1—C6A	−12.7 (6)	O1^i^—Br—C14—C13	−44.1 (5)
N3—N2—C1—C6B	28.2 (10)	C13—C14—C15—C16	1.3 (6)
N2—N3—C4—O1	−175.0 (3)	Br—C14—C15—C16	−179.8 (3)
N2—N3—C4—C5	2.1 (6)	C14—C15—C16—C11	−0.3 (6)
O1—C4—C5—C6B	164.1 (11)	C12—C11—C16—C15	−0.8 (5)
N3—C4—C5—C6B	−13.0 (11)	C1—C11—C16—C15	178.0 (3)
O1—C4—C5—C6A	−157.3 (5)	C6B—C5—C20—C21	−2.8 (14)
N3—C4—C5—C6A	25.7 (6)	C6A—C5—C20—C21	−46.4 (8)
O1—C4—C5—C20	−14.0 (6)	C4—C5—C20—C21	174.9 (4)
N3—C4—C5—C20	168.9 (4)	C5—C20—C21—C22	−87.0 (5)
C20—C5—C6A—C1	175.7 (5)	C5—C20—C21—C26	88.7 (5)
C4—C5—C6A—C1	−44.3 (9)	C26—C21—C22—C23	−1.5 (6)
N2—C1—C6A—C5	40.7 (10)	C20—C21—C22—C23	174.3 (4)
C11—C1—C6A—C5	−152.3 (5)	C21—C22—C23—C24	−0.2 (7)
C20—C5—C6B—C1	−154.1 (8)	C22—C23—C24—C25	2.0 (7)
C4—C5—C6B—C1	28.1 (19)	C22—C23—C24—C27	−177.2 (4)
N2—C1—C6B—C5	−38.1 (19)	C23—C24—C25—C26	−2.1 (6)
C11—C1—C6B—C5	169.9 (10)	C27—C24—C25—C26	177.1 (4)
N2—C1—C11—C12	21.7 (5)	C22—C21—C26—C25	1.5 (6)
C6A—C1—C11—C12	−145.8 (6)	C20—C21—C26—C25	−174.3 (4)
C6B—C1—C11—C12	175.0 (11)	C24—C25—C26—C21	0.3 (7)
N2—C1—C11—C16	−157.2 (4)	C28—O3—C27—O2	1.4 (7)
C6A—C1—C11—C16	35.4 (7)	C28—O3—C27—C24	−178.3 (4)
C6B—C1—C11—C16	−3.8 (11)	C25—C24—C27—O2	173.6 (5)
C16—C11—C12—C13	1.0 (5)	C23—C24—C27—O2	−7.2 (7)
C1—C11—C12—C13	−177.9 (3)	C25—C24—C27—O3	−6.6 (6)
C11—C12—C13—C14	0.0 (6)	C23—C24—C27—O3	172.6 (4)
C12—C13—C14—C15	−1.2 (6)		

Symmetry codes: (i) *x*−2, *y*−1, *z*.

### Hydrogen-bond geometry (Å, °)

**Table:**

*D*—H···*A*	*D*—H	H···*A*	*D*···*A*	*D*—H···*A*
N3—H3···O1^ii^	0.86	2.08	2.910 (4)	162
C14—Br···O1^i^	1.901 (3)	3.096 (2)	?	152.32 (12)

Symmetry codes: (ii) −*x*+3, −*y*+1, −*z*+1; (i) *x*−2, *y*−1, *z*.

## References

[bb1] Abudshait, S. A. (2007). *Molecules*, **12**, 25–42. 10.3390/12010025 PMC6149388 17693951

[bb2] Altomare, A., Burla, M. C., Camalli, M., Cascarano, G. L., Giacovazzo, C., Guagliardi, A., Moliterni, A. G. G., Polidori, G. & Spagna, R. (1999). *J. Appl. Cryst.* **32**, 115–119.

[bb3] Barbaro, R., Betti, L., Botta, M., Corelli, F., Giannaccini, G., Maccari, L., Manetti, F., Strappaghetti, G. & Corsano, S. (2001). *J. Med. Chem.* **44**, 2118–21332. 10.1021/jm010821u 11405649

[bb4] Cao, S., Qian, X., Song, G., Chai, B. & Jiang, Z. (2003). *J. Agric. Food Chem.* **51**, 152–155. 10.1021/jf0208029 12502400

[bb5] Coelho, A., Raviña, E., Fraiz, N., Yáñez, M., Laguna, R., Cano, E. & Sotelo, E. (2007). *J. Med. Chem.* **50**, 6476–6484. 10.1021/jm061401d 18031002

[bb6] Dogruer, D. S., Sahin, M. F., Kupeli, E. & Yesilada, E. (2003). *Turk. J. Chem.* **27**, 727–738.

[bb7] Enraf–Nonius (1989). *CAD-4 Software* Enraf–Nonius, Delft, The Netherlands.

[bb8] Giovannoni, M. P., Cesari, N., Vergelli, C., Graziano, A., Biancalani, C., Biagini, P., Ghelardini, C., Vivoli, E. & Piaz, V. D. (2007). *J. Med. Chem.* **50**, 3945–3953. 10.1021/jm070161e 17629262

[bb9] Katrusiak, A. & Baloniak, S. (1994). *Tetrahedron*, **50**, 12933–12940.

[bb10] Macrae, C. F., Bruno, I. J., Chisholm, J. A., Edgington, P. R., McCabe, P., Pidcock, E., Rodriguez-Monge, L., Taylor, R., van de Streek, J. & Wood, P. A. (2008). *J. Appl. Cryst.* **41**, 466–470.

[bb11] Malinka, W., Redzicka, A. & Losach, O. (2003). *Il Farmaco*, **59**, 457–462. 10.1016/j.farmac.2004.03.002 15178308

[bb12] Meyer, E., Joussef, A. C., Gallardo, H. & de Souza, L. de B. P. (2004). *Synth. Commun.* **34**, 783–793.

[bb13] North, A. C. T., Phillips, D. C. & Mathews, F. S. (1968). *Acta Cryst.* A**24**, 351–359.

[bb14] Piaz, V. D., Ciciani, G. & Giovannoni, M. P. (1994). *Synthesis*, pp. 669–671.

[bb15] Pieretti, S., Dominici, L., Giannuario, A. D., Cesari, N. & Piaz, V. D. (2006). *Life Sci.* **79**, 791–800. 10.1016/j.lfs.2006.02.026 16546218

[bb16] Sayed, G. H., Sayed, M. A., Mahmoud, M. R. & Shaaban, S. S. (2002). *Egypt. J. Chem.* **45**, 767–776.

[bb17] Sheldrick, G. M. (2008). *Acta Cryst.* A**64**, 112–122. 10.1107/S0108767307043930 18156677

[bb18] Spek, A. L. (1996). *HELENA* University of Utrecht, The Netherlands.

[bb19] Spek, A. L. (2009). *Acta Cryst.* D**65**, 148–155. 10.1107/S090744490804362X PMC2631630 19171970

[bb20] Vergelli, C., Giovannoni, M. P., Pieretti, S., Giannuario, A. D., Piaz, V. D., Biagini, P., Biancalani, C., Graziano, A. & Cesari, N. (2007). *Bioorg. Med. Chem.* **15**, 5563–5575. 10.1016/j.bmc.2007.05.035 17548197

[bb21] Voronina, Yu. K., Saifina, L. F., Romanova, E. S., Lodochnikova, O. A. & Litvinov, I. A. (2009). *J. Struct. Chem.* **50**, 588–591.

[bb22] Wexler, R. R., Greenlee, W. J., Irvin, J. D., Goldberg, M. R., Prendergast, K., Smith, R. D. & Timmermans, P. B. M. W. M. (1996). *J. Med. Chem.* **39**, 625–656. 10.1021/jm9504722 8576904

[bb23] Xu, H., Hu, X.-H., Zou, X.-M., Liu, B., Zhu, Y.-Q., Wang, Y., Hu, F.-Z. & Uang, H.-Z. (2008). *J. Agric. Food Chem.* **56**, 6567–6572. 10.1021/jf800900h 18605735

[bb24] Zhang, C.-T., Wu, J.-H., Zhou, L.-N., Wang, Y.-L. & Wang, J.-K. (2006). *Acta Cryst.* E**62**, o2999–o3000.

[bb25] Zhou, Z.-Z. & Zhou, H.-B. (2007). *Acta Cryst.* E**63**, o2512.

